# A Case Report of Tuberculosis in the Finger

**DOI:** 10.7759/cureus.14071

**Published:** 2021-03-23

**Authors:** Elham Mahdi, Lulwah Alabdan, Sadiq Amer, Mais B Alashqar, Sami Almustanyir

**Affiliations:** 1 Internal Medicine, Prince Mohammed Bin Abdulaziz Hospital, Riyadh, SAU; 2 Pathology, Prince Mohammed Bin Abdulaziz Hospital, Riyadh, SAU; 3 College of Medicine, Alfaisal University, Riyadh, SAU; 4 Medicine, Ministry of Health, Riyadh, SAU

**Keywords:** tuberculosis, osteomyelitis, cutaneous tuberculosis

## Abstract

Tuberculosis is a chronic, infectious disease that predominantly affects the respiratory system. Of the least common extrapulmonary forms of the disease is cutaneous tuberculosis. We present an unusual case, which is of cutaneous tuberculosis in the finger, manifesting as an ulcerating, erythematous lesion. This had later spread to the adjacent soft tissue and bones, causing osteomyelitis of the phalanges. It is important that physicians maintain a high index of suspicion when faced with atypical skin lesions to avoid the sequelae of the local and disseminated spread of a tuberculosis infection.

## Introduction

Tuberculosis (TB) is a chronic infection that is caused by the bacterium *Mycobacterium tuberculosis*, in the majority of cases. This disease is well known for creating caseating granulomas and an indolent inflammatory response, most commonly in the respiratory system. It can, however, localize in other organs. Of the recognized forms of the disease, cutaneous TB is one of the least common, accounting for only 0.5-2% of all extrapulmonary TB cases [1]. We present an exceedingly unusual manifestation of the disease.

## Case presentation

A 33-year-old lady from the Philippines presented to our hospital with a left ring finger lesion for the past one month. It had initially started as an area of erythema, then a small papule developed. The papule then continued to progress into a large, painless swelling, measuring 2 x 3 cm. The lesion is located in the lateral aspect of the middle phalanx of the ring finger. The patient had no other skin lesions or rashes on her body. Additionally, this was the first time that she had presented with such a swelling. There is no incident or injury that preceded this finger swelling. Furthermore, she reported no fever, chills, rigors, night sweats, fatigue, or dizziness.

She had sought medical attention for this by visiting her primary care provider, and was prescribed two courses of empirical antibiotics, with no significant improvement.

Our patient is otherwise healthy, with no known chronic diseases, infections, immune disorders, or such. She works as a housemaid, and no similar condition occurred in her own family or in the family that she currently works for. She has had no contact with sick patients, and no recent travels. There was also no contact with animals. Her systemic review was unremarkable.

**Figure 1:**
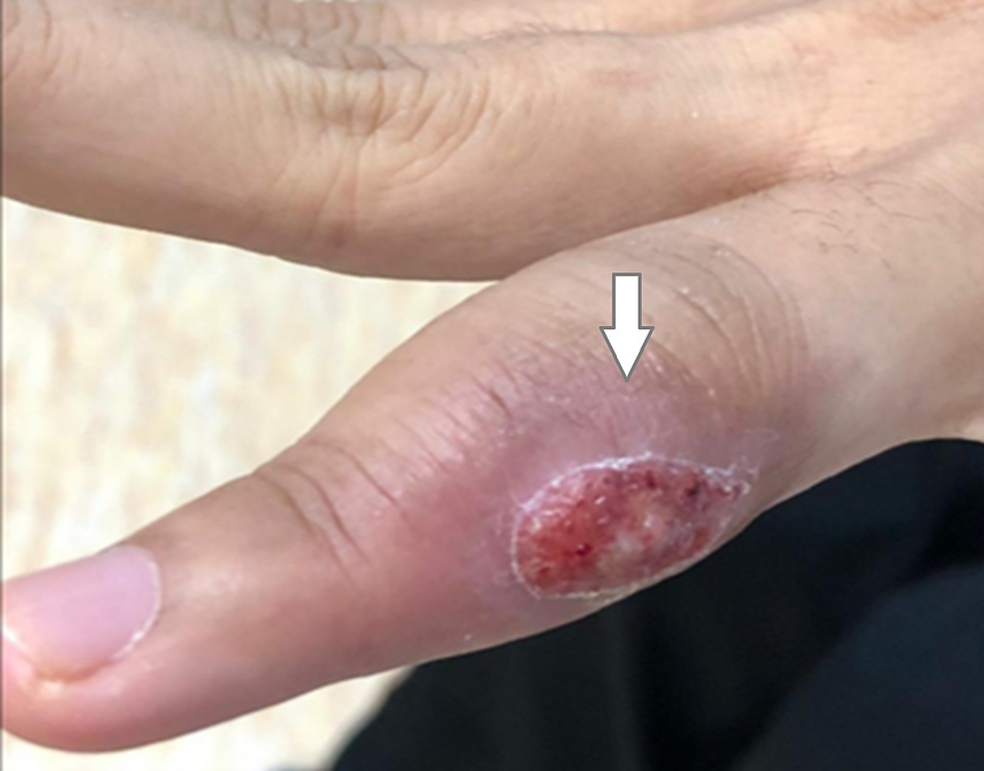
Left ring finger with an ulcerated swelling and erythema

Her vitals were stable and within normal limits. Local examination revealed an ulcerated swelling, surrounded by erythema, over the middle phalanx of the left ring finger (Figure 1). A minimal amount of pus was expelled when pressure was applied to the area. Moreover, mild tenderness was noted on deep palpation and we found limited movement at the proximal interphalangeal joint. The skin of the rest of this hand and the other hand were both completely normal. Additionally, examination of the other joints of the hand was also normal. There was intact sensation and a strong distal pulse. No regional lymph node enlargement was found. Respiratory, cardiovascular, abdominal, and neurological examinations were unremarkable.

Laboratory tests were notable for normocytic normochromic anemia, with a hemoglobin of 10 g/dL (normal range for females: 12-15.5 g/dL). There was also an elevated erythrocyte sedimentation rate of 120 mm/h (normal range: 0-17 mm/h). C-reactive protein level was 5.3 mg/L (normal range: <10 mg/L).

A finger X-ray was done and showed osteolysis of the proximal phalanx and of the base of the distal phalanx. The chest X-ray was normal. Ultrasound of the finger showed lobulated collections at the site of the lesion.

Upon further testing, the patient had a positive purified protein derivative (PPD) test. She was also tested for human immunodeficiency virus using enzyme-linked immunosorbent assay, and the result was negative.

To manage the lesion, irrigation and debridement were done under local anesthesia. A moderate volume of pus was extracted and samples were taken for histopathology and tissue culture. The patient was then started on empirical intravenous antibiotics until the diagnosis could be confirmed, but she showed no clinical improvement.

**Figure 2:**
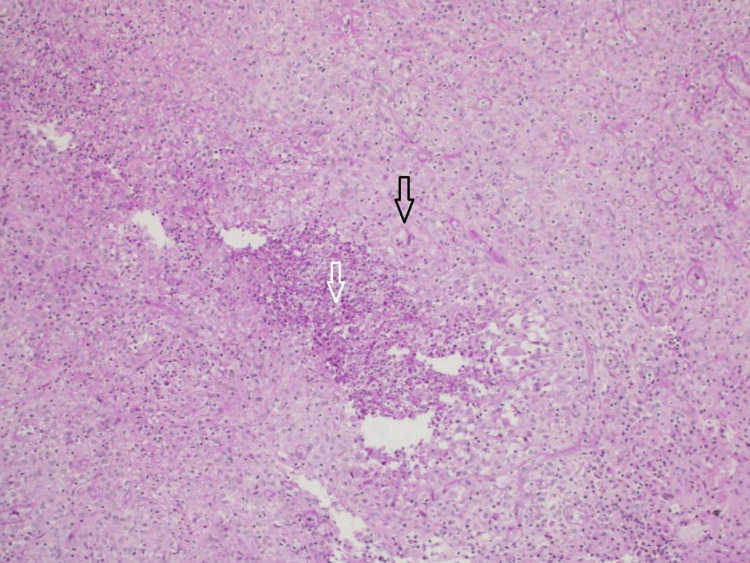
Microscopic examination of the finger lesion biopsy showing intense granulomatous inflammation (black arrow) in the reticular dermis and subcutaneous tissue with foci of central necrosis (white arrow)

The tissue culture was found negative. The samples came back positive on acid-fast bacilli (AFB) smear, and positive for tuberculosis polymerase chain reaction (TB PCR). The histology of the biopsy displayed intense granulomatous inflammation in the reticular dermis and subcutaneous tissue with foci of central necrosis (Figure 2). The sample had no microscopic evidence of other bacterial or fungal infections.

During her hospital course, the patient then started to develop a painful ulcer on the right foot. A sample of the discharge from that ulcer was taken and was positive for TB PCR as well.

Hence, a diagnosis of cutaneous TB with osteomyelitis was confirmed.

The first-line anti-TB regimen (consisting of isoniazid, rifampicin, pyrazinamide, and ethambutol) was immediately started for this patient, and she was discharged from the hospital for out-patient follow-up.

Because of the COVID-19 pandemic and the transportation restrictions at that time, we lost follow-up with her in our hospital, and then she returned to the Philippines.

## Discussion

As per the World Health Organization, the incidence of TB worldwide in 2019 was approximately 10 million new cases [2]. Additionally, 1.2 million patients died of TB in that year [2]. These vast numbers are mainly concentrated in developing countries, namely India, Indonesia, and China [2]. This infection is also more prevalent in those with acquired immunodeficiency disease [2]. Extrapulmonary TB, in particular, is more common in females, HIV-positive patients, and in non-Hispanic blacks [3,4]. When a disease is this encompassing, it becomes critical for physicians to be familiar with even the least common manifestations of it.

Of the extrapulmonary sites, cutaneous TB represents only a minority of the cases, at <2% [1]. Many physicians may have not even heard of it. It is, therefore, less likely to be considered as a differential diagnosis when presented with an erythematous or ulcerating lesion of the skin, as we found in our patient. Moreover, cutaneous TB can have a plethora of presentations, each with its own list of differential diagnoses and mimickers. Cutaneous TB can be the primary lesion, presenting as tuberculosis verrucosa cutis, tuberculous chancre, or tuberculous lupus [5]. It can also be secondary to systemic spread of the disease, presenting as tuburculids, tuberculous lupus vulgaris, acute cutaneous miliary TB, colliquative TB, or orificial TB [5]. This makes the diagnosis much more challenging, yet it remains crucial to catch, as the progression of cutaneous TB can be the spread to and destruction of the adjacent structures. In our patient, the disease had progressed to osteomyelitis of the phalanges of the finger. She is a maid and needs full function of her hands to perform her job.

Additionally, of the cutaneous TB sites, the finger is seldom seen. It has been identified in only a small number of cases in adults [6-8]. There have been, however, more reports of TB tenosynovitis or dactylitis in the hands, with swellings and pain, rather than cutaneous lesions [9-16].

When suspecting a cutaneous TB infection from the history, risk factors, and physical examination of the patient, the diagnostic tests available are PPD, histology (with an AFB smear), culture, and PCR [17]. The initial screening is usually done by PPD, but depending on the type of cutaneous TB lesion, the test may or may not be positive, even if the infection has originated from an endogenous route [17]. As for the histological staining, it is an excellent tool; however, it requires the lesion to have a high bacterial load to increase the chance that the microbes will be visualized [17]. The cultures are the definitive diagnostic test, but they may take six to eight weeks for *M. tuberculosis* to be isolated [17]. PCR testing is currently one of the best options, as it can detect the presence of the mycobacterium quickly, and even with a low bacillary load in the lesion. As PCR testing directly identifies specific DNA sequences using tailor-made probes, it has a high sensitivity and specificity as well [17].

Once the diagnosis has been confirmed, the treatment is similar to that of pulmonary TB. The standard regimen, as per The Centers for Disease Control and Prevention, is a two-month course of rifampicin, isoniazid, pyrazinamide, and ethambutol, followed by four months of only rifampicin and isoniazid. Surgical excision, cryotherapy, and electrocautery may be used as adjuncts to the medical therapy [17]. HIV-positive patients may require more specialized and more aggressive treatments, as they are more likely to present with multi-drug-resistant mycobacteria [17]. 

## Conclusions

Dermatologists and primary physicians need to maintain a high index of suspicion for cutaneous TB, especially when a patient is presenting with an unusual, chronic lesion, which cannot be explained otherwise. It may present in patients with no history of TB exposure, no previous medical history, and no other symptoms. Initial testing may be done using PPD and AFB smears; however, PCR remains the most accurate and time-efficient option to identify the mycobacterium. A long-term treatment regimen would be required in cases of cutaneous TB, including eight weeks of rifampicin, isoniazid, pyrazinamide, and ethambutol, followed by 16 weeks of isoniazid and rifampicin only.
